# Tilt after-effect from high spatial-frequency patterns in the amblyopic eye of adults with anisometropic amblyopia

**DOI:** 10.1038/srep08728

**Published:** 2015-03-04

**Authors:** Jiawei Zhou, Lin Li, Pan Zhang, Jie Xi, Yifeng Zhou, Zhong-Lin Lu, Chang-Bing Huang

**Affiliations:** 1 Key Laboratory of Behavioral Science, Institute of Psychology, Chinese Academy of Sciences, 16 Lincui Road, Chaoyang Dist., Beijing, 100101, China; 2 CAS Key Laboratory of Brain Function and Disease, and School of Life Sciences, University of Science and Technology of China, 230027, Hefei; 3 Laboratory of Brain Processes (LOBES), Center for Cognitive and Brain Sciences, Departments of Psychology, The Ohio State University, Columbus, OH, 43210, United States of America

## Abstract

With abnormal visual cortical development, amblyopia is generally associated with high spatial frequency deficits in spatial vision. In this study, we aim to answer a critical question: How much high spatial frequency information is available to the amblyopic visual system? We measured the tilt after-effect following adaption to perceptually resolvable and unresolvable sinewave gratings, and showed that gratings with spatial frequency up to 1.5 times the cutoff frequency in grating orientation identification can still produce significant tilt after-effects in adults with amblyopia. Our results suggest that neural connections in the amblyopic visual cortex, at least in V1, may have profoundly developed to represent high spatial frequency information. The demonstration of extant neural connections for high spatial frequencies may have important implications for the development of training protocols for amblyopia treatment. Our paradigm may also serve as a non-invasive probe to diagnose the status of neural connections in other visual deficits.

Early abnormal visual experience can disrupt visual development and cause amblyopia^1^, one of the leading causes of monocular vision loss that affects 3% of the population^2^. Generally acknowledged as a spatial vision deficit in high spatial frequencies^3^,^4^, amblyopia has become a test case for understanding developmental plasticity^5^. Traditional occlusion therapy can recover high spatial frequency vision for younger children but fails in older children and adults, suggesting that high spatial frequency information might be absent in the adult amblyopic visual system. On the other hand, recent evidence from perceptual learning studies indicates that vision in high spatial frequency can be at least partially recovered in adults with amblyopia^6^,^7^,^8^,^9^. An intriguing question is: How much high spatial frequency information is available to the amblyopic visual system? The answer to this question could be extremely important for understanding cortical processing in amblyopic vision and may have important implications for the development of training protocols for amblyopia treatment.

Although animal studies found selective sensitivity loss of cortical cells tuned to high spatial frequencies in amblyopic vision^10^,^11^,^12^, combined psychophysical measurements and electrophysiological single-unit recordings in monkey have concluded that the degree of neuronal response abnormality in V1 or MT was smaller than the observed behavioral deficits^5^,^13^, suggesting additional deficits in higher visual areas^14^. The accumulating evidence leads to the surprising possibility that high spatial frequency information may be represented in early visual areas. Traditional behavioral measures, including visual acuity and contrast sensitivity function, do not directly measure cortical representation of visual information because performance in those tasks is determined by the signal to noise ratio in the visual system^15^,^16^. High spatial frequency deficits documented by visual acuity and contrast sensitivity function might reflect high internal noise rather than the absence of high spatial frequency information in the amblyopic visual system.

Exposure to visual patterns of high contrast creates after-effects in perception. The orientation of a test pattern slightly tilted relative to the pre-exposed pattern may be perceptually exaggerated, i.e. the so-called tilt after-effect (TAE)^17^,^18^. He and MacLeod (2001) found that TAE occurred even when the pre-exposed grating was too fine to be perceptually resolved, revealing information that is represented neurally without conscious awareness. Here, we measured the tilt after-effects after amblyopic observers were exposed to perceptually unresolvable high spatial frequency gratings, in an attempt to bypass internal noise limitations and to examine whether perceptually unresolvable gratings are represented in the amblyopic visual cortex. The results allowed us to reveal how much high spatial frequency information is available to the amblyopic visual system.

## Results

**Figure 1:**
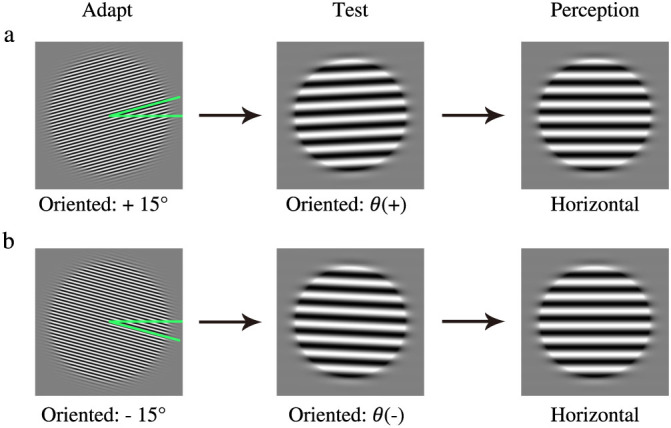
The tilt after-effect (TAE). (a) Adapting to a high-contrast grating tilted +15° from horizontal rendered a grating tilted *θ(+)* perceptually horizontal. (b) Adapting to a high-contrast grating tilted −15° from horizontal rendered a grating tilted *θ(−)* perceptually horizontal. The TAE threshold is quantified as the mean of *θ(+)* and *θ(−)* to eliminate potential orientation bias.

In the experiment, we used the method of constant stimuli to quantify the magnitude of TAE following exposures to high-contrast (100%) gratings titled either +15° or −15° from horizontal. The orientation of the 4 cycle/° test grating was varied to determine the orientation [*θ(+)* and *θ(−)* for the +15° and −15° conditions, respectively] at which the test is perceived as being horizontal (Figure 1). To eliminate any potential orientation bias, TAE threshold was quantified as the mean of *θ(+)* and *θ(−)*.

**Figure 2:**
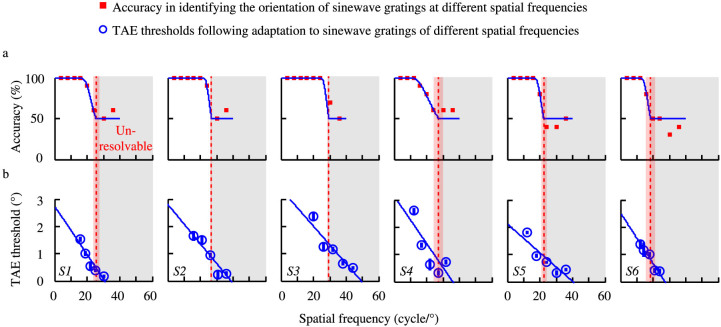
Orientation identification accuracy and TAE thresholds for the amblyopic eye of six amblyopic observers. Each panel represents results from one observer. (a) The ‘
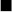
’ symbols represent the accuracy in identifying the orientation of high contrast sinewave gratings. The red dashed line indicates the cutoff spatial frequency in grating orientation identification, defined as the lowest spatial frequency associated with chance performance in orientation identification. The standard error of the cutoff spatial frequency was estimated using a bootstrap procedure with 500 resampling repetitions and is marked as a pink rectangle area surrounding the red dashed line in each panel. The gray rectangle area marks the range of unresolvable spatial frequencies. (b) The ‘
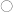
’ symbols represent TAE thresholds following adaptation to sinewave gratings of different spatial frequencies. The error bars represent standard errors estimated from a bootstrap procedure. The blue solid lines represent the best linear fits to the TAE threshold vs. spatial frequency functions. The x-intercepts of the blue lines indicate the cutoff spatial frequencies in generating TAE.

Five adapting spatial frequency conditions, covering sinewave gratings that were easily visible to those that were completely unresolvable, were selected for each observer based on the individual's orientation identification performance (Figure 2a, red curves). A sinewave grating is defined as unresolvable if its spatial frequency is higher than the cutoff spatial frequency in grating orientation identification (see **Methods**). The TAE thresholds for six anisometropic amblyopes are shown in Figure 2b (characteristics of the observers can be found in Table 1). Adapting to gratings with resolvable spatial frequencies (blue dots on the white background in each panel) yielded an average TAE threshold of 1.35 ± 0.14° (Mean ± S.E.), consistent with previous reports in normal observers^18^. What is surprising is that adapting to gratings of unresolvable spatial frequencies (blue dots on the gray background) still yielded considerable TAE with an average effect size of 0.48 ± 0.07°. Although the TAE thresholds following adaptation to unresolvable gratings were smaller than those following adaptation to resolvable gratings (Independent samples t-test, t(28) = 5.35, *p* = 0.00001), a Z-test revealed that all the TAE thresholds were significantly greater than 0 (*p* < 0.05, see Table 2 for Z-scores).

**Figure 3:**
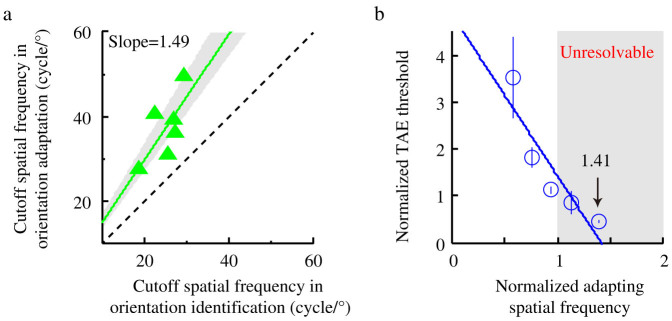
The relationship between TAE and orientation identification. (a) Cutoff spatial frequency in generating TAE as a function of cutoff spatial frequency in orientation identification. Each ‘
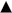
’ symbol represents data from one observer. The green solid line is the linear regression to the data with a slope of 1.49, indicating that the cutoff spatial frequency in orientation adaptation is about 1.49 times that in orientation identification. The gray area covers the 95% confidence interval of the regression model. The lower black dashed line is the identity line (slope = 1). (b) Average normalized TAE threshold as a function of normalized adapting spatial frequency. The adapting spatial frequency was normalized to the cutoff spatial frequency in orientation identification. The magnitude of the TAE was normalized to TAE threshold at the orientation identification cutoff frequency. The ‘
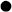
’ symbols represent data averaged across observers. The gray rectangle area marks the range of unresolvable spatial frequencies. The blue line represents the best-weighted linear fit to the data. The x-intercept of the best fitting linear model indicates the normalized cutoff spatial frequency in generating TAE. Error bars indicate S.E.M.

We fit a linear regression model to the TAE threshold vs. spatial frequency data in Figure 2. It is clear that the cutoff spatial frequencies for generating TAE, i.e., the x-intercepts of the linear regression model, are significantly higher than those for grating orientation identification, as signified by the positions of the red dashed lines in Figure 2. The cutoff spatial frequencies in orientation identification and in the generation of TAE are significantly correlated (Figure 3a; R = 0.946, *p* = 0.001), with a slope of 1.49. Averaged across observers, the cutoff spatial frequency is 37.21 ± 3.19 cycle/° (Mean ± S.E.) for generating TAE and 24.96 ± 1.60 cycle/° for grating orientation identification; the ratio of the cutoff spatial frequencies in these two tasks is 1.49 ± 0.09. To further verify the relationship, we normalized the spatial frequency axis of the TAE threshold vs. spatial frequency function by the cutoff spatial frequency in orientation identification and the TAE threshold by that at the orientation identification cutoff frequency, and then averaged the results across observers (Figure 3b; see **Methods** for details). A weighted linear regression resulted in an x-intercept of 1.41, perfectly matching the average ratio of the two types of cutoff spatial frequencies.

## Discussion

Our study demonstrated that adapting to spatial frequencies up to about 1.5 times the cutoff frequency for grating orientation identification could still produce significant tilt-after effect. The results showed clearly that the amblyopic visual cortex is capable of representing gratings with spatial frequencies up to 1.5 times the cutoff frequency for grating orientation identification.

The estimated 1.5× ratio is comparable to those reported by He and MacLeod (2001) who found ratios of 1.4 and 1.2 in two adults with normal vision. We should point out that the cutoff spatial frequency derived from the tilt after-effect in the current study might be an underestimate of the ability of the amblyopic vision system in representing high spatial frequency information. This is because we didn't bypass the imperfect optics of the eyes^19^, which attenuates high spatial frequency information before neural processing^20^. Possible solutions to the problem include measuring TAE to perceptually unresolvable gratings after higher-order aberration corrections^20^ or using interference fringe patterns^17^. On the other hand, without these highly elaborated procedures, our measurements reflect what is represented in the visual cortex of observers with amblyopic vision in their natural viewing conditions.

One technical concern is the way we derived cutoff spatial frequency in identifying orientation with a stimulus duration of 250 ms, which might underestimate the cutoff frequency since observers may have better orientation discriminability with longer grating presentations, e.g., 60 seconds, as in the TAE measurement. This issue was addressed in a pilot study (supplementary Table S1 and Figure S1), in which we estimated the cutoff spatial frequency in orientation discrimination with both 250 ms and unlimited stimulus durations. Five amblyopes were tested. The estimated cutoff spatial frequencies in the two conditions were not significantly different (2-tailed paired sample t-test, t(4) = −1.25, *p* = 0.28.) Based on the results from the pilot study, we decided to use the 250-ms stimulus duration to save test time and to avoid potential adaptation effects in orientation identification. Nevertheless, we need to emphasize that the cutoff SF in orientation discrimination, which we measured here with a 250-ms stimulus duration, might be slightly less than but not significantly different from that obtained with a longer stimulus duration (Supplementary Materials). The underestimation would not change the main conclusion but may slightly change the magnitude of the difference between TAE and orientation discrimination cutoff frequencies. If we use the ratio between cutoff spatial frequencies obtained in short and long stimulus durations with a different group of subjects as a correction factor (Supplementary materials), the ratio between the TAE and orientation discrimination cutoff spatial frequencies would be about 1.2 instead of 1.4.

Because the primary visual cortex (V1) is the earliest stage of visual processing with orientation selectivity^21^, our findings indicate that neural connections in the amblyopic cortex, at least in V1, may have profoundly developed to represent high spatial frequency information, far beyond what we would expect from traditional spatial vision measurements. Interestingly, the amblyopic brain doesn't have conscious access to the representation of such high spatial frequency information. We speculate that high internal noise, non-optimal perceptual template, and/or jittered topological projections^22^,^23^,^24^,^25^ dramatically degraded the quality of such representation (i.e. lowering signal to noise ratio) and thus obstructed the signal from conscious perception in amblyopic vision. In the current study, we took advantage of the fact that the TAE paradigm involves a supra-threshold orientation judgment, which relied mostly on cortical adaptation to the adapting signal grating.

Our results may also have important implications for the development of training protocols for amblyopia treatment. The demonstration of extant neural connections for high spatial frequencies is consistent with results from many perceptual learning studies that showed high spatial frequency deficits in amblyopia could be at least partially recovered in adults with amblyopia^6^,^7^,^8^,^9^,^26^. Huang et al (2010) showed internal noise reduction played an important role in perceptual learning in amblyopia. In future studies, it would be interesting to investigate whether the extant neural connections for high spatial frequencies involved in adaptation can be exploited through internal noise reduction in perceptual learning to improve amblyopic vision.

The demonstration of extant neural connections for high spatial frequencies gives us a glimpse of cortical processing and in particular representation of high spatial frequency in amblyopic vision. We should note that all of our observers are adults with mild to moderate anisometropic amblyopia, thus our observation may or may not generalize to more severe or other types of amblyopia. Nevertheless, the successful application of the TAE paradigm in this study suggests that it may also serve as a non-invasive probe to diagnose the status of neural connections in other clinical conditions.

## Methods

### Participants

Six teenage or adult anisometropic amblyopes (average age: 22.3 ± 2.6 yrs; 2 females), with mild to moderate amblyopia (visual acuity, 20/30–20/75), participated in the study. Characteristics of the observers are listed in Table 1. All observers were naive to the purpose of the experiment. Informed consent was obtained prior to the study, which was approved by the Institutional Review Board of the University of Science and Technology of China, and the Institutional Review Board of the Institute of Psychology, Chinese Academy of Science. The methods were carried out in accordance with the approved guidelines.

### Apparatus

All measurements were conducted on a PC computer running Matlab (MathWorks, Inc.) with PsychToolBox 2.54 extensions^27^,^28^. The stimuli were presented on a Sony G220 Trinitron monitor with a 2048 × 1536 resolution and a 60 Hz refresh rate. The background luminance was 31.2 cd/m^2^. High grayscale resolution (i.e. 14 bit) was achieved using a VideoSwitcher (http://lobes.osu.edu/videoSwitcher/). Gamma correction was conducted using a psychophysical procedure in combination with a photometer^29^.

In the experiment, the observer placed his/her head on a chinrest and viewed the stimuli with the amblyopic eye. The fellow eye was occluded with an opaque patch. Stimuli were sinewave gratings at eight spatial frequencies: 4, 8, 12, 16, 20, 24, 30 and 36 cycle/°. They were presented with 100% contrast in a 3° diameter circular window at a viewing distance of 350 cm. A 0.46° half-Gaussian ramp was applied to the edge of the circular window to minimize edge effects.

### Experimental design

The accuracy of grating orientation identification (tilted +15° or −15° from horizontal) was measured with 50 trials in each of the eight spatial frequency conditions, with a total of 400 trials. The cutoff spatial frequency, defined as the lowest spatial frequency associated with chance performance (50% accuracy) in orientation identification, was determined by the following function:
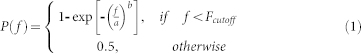
where *P(f)* is the measured fraction correct in orientation identification, the two free parameters, *a* and *b*, define the shape of the psychometric function. The cutoff spatial frequency can be computed as:
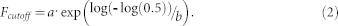
The spatial frequencies of the adapting sinewave gratings were chosen for each observer based on individual performance in the orientation identification task. In general, 1 or 2 easily resolvable (identification accuracy > = 0.90), 1 resolvable (accuracy < 0.90), and 2 to 3 unresolvable spatial frequencies, corresponding to spatial frequencies much lower than, lower than, and higher than the cutoff spatial frequency in grating orientation identification, were chosen.

To estimate the magnitude of TAE, observers were asked to judge the orientation of the 4 cycle/° test grating (clockwise or counter-clockwise from horizontal) following adaptation. Five test orientations were used in each adaptation condition based on results from pilot studies. Each observer completed a total of 5 (adapting spatial frequencies) × 2 (adapting orientations) × 5 (test orientations) × 25 (trials) = 1250 trials. The trials were blocked according to the spatial frequency and orientation of the adapting grating. The orientations of the test gratings were randomized in each block. The measured probability (of being perceived as counter-clockwise from horizontal) versus test grating orientation curve was fitted by a cumulative Gaussian distribution function (*normcdf* in Matlab). The 50% point of the best fitting Gaussian function, i.e., point of subjectively equality (PSE) at which the test grating was perceived as horizontal, was defined as the TAE threshold. We obtained 10 TAE thresholds for sinewave gratings at 5 adapting spatial frequencies, each at 2 adapting orientations. To eliminate orientation bias, the final TAE threshold in each adapting spatial frequency condition was quantified by averaging TAE thresholds in the two adapting orientation conditions. The standard errors of the TAE thresholds were derived using a bootstrap procedure^30^.

### Procedures

#### (1) Visibility of adapting sinewave gratings

For each trial, a sinewave grating, with one of eight spatial frequencies and tilted either +15° or −15° from horizontal, was presented in the center of the display for 250 ms. Observers were instructed to report the orientation of the grating by pressing one of two keys on the computer keyboard. Note that a 250-ms presentation, rather than a longer duration, e.g., 60 s, was used in the test to save time and to avoid potential adaptation effects on orientation identification. However, our pilot study showed that the estimated cutoff spatial frequencies in the 250 ms and unlimited duration conditions were not significantly different (see supplementary Figure S1 for details).

#### (2) Magnitude of the TAE

To avoid any potential interactions between different adaptation conditions, we measured TAE thresholds following adaptation to sinewave gratings at five spatial frequencies and two orientations in ten separate blocks, with a single adaptation condition in each block. Within each block, observers first adapted to a sinewave grating for 60 seconds, during which they were instructed to fixate at the central fixation point even if they could not see the grating. To minimize contrast adaptation, the adapting grating was phase-reversed at 4 Hz. A beep and a blank screen at mean luminance were provided at the end of the 60-sec preparation adaptation, which also signaled the beginning of the TAE measurement. In each TAE test trial, observers first adapted to the sinewave grating for 5 seconds (top-off). They were then presented with a 250-ms blank screen at mean luminance and a test grating for 250 ms. The test grating was always at 100% contrast and at 4 cycle/°, and had the same size and Gaussian envelope as that of the adapting grating. Observers were instructed to report the orientation of the test grating (clockwise or counter-clockwise from horizontal) by pressing one of two keys on the computer keyboard. The next trial started immediately after the response. Observers took a (at least) 5-minute mandatory break between blocks.

### Data analysis

The average normalized TAE threshold vs. normalized adapting spatial frequency function was derived using the following procedure:

 1) The cutoff spatial frequency, corresponding to the lowest spatial frequency associated with chance performance in orientation identification, was computed by fitting Eq.1 to the data from each observer.

 2) The TAE threshold at the cutoff spatial frequency in orientation identification was computed by linear interpolation of the TAE vs. spatial frequency curve for each observer.

 3) The TAE thresholds at the five adapting spatial frequencies were normalized to the TAE threshold at the cutoff spatial frequency in orientation identification. The adapting spatial frequencies were normalized to the cutoff spatial frequency in orientation identification.

 4) The normalized adapting spatial frequencies were then divided into five bins: [0.4,0.65), [0.65,0.85), [0.85,1), [1,1.2), and [1.2,1.7). Data within these five bins were averaged and weighted by their variance.

## Author Contributions

J.Z., C.-B.H., Y.Z. and Z.-L.L. designed research; J.Z., L.L., P.Z. and J.X. performed research; J.Z. and C.-B.H. analyzed data; and J.Z., C.-B.H. and Z.-L.L. wrote the paper.

## Supplementary Material

Supplementary Information Supplementary Information

## Figures and Tables

**Table 1:** Characteristics of the amblyopic observers. Abbreviations: AE = amblyopic eye; FE = fellow eye; DS = diopters sphere; DC = diopters cylinder; MAR = minimum angle of resolution

Observer ID	Age (yr)	Sex	Eye	Correction	Visual acuity† (LogMAR)
S1	23	Male	AE	+0.50DS/+1.00DC × 60°	0.19
			FE	−3.25DS/−0.50DC × 15°	−0.25
S2	23	Male	AE	+3.00DS/+0.50DC × 90°	0.37
			FE	−0.50DS/−0.50DC × 5°	0.18
S3	23	Female	AE	+0.50DS/+0.50DC × 95°	0.18
			FE	−3.00DS	−0.03
S4	17	Male	AE	+2.50DS	0.57
			FE	−3.00DS	0.07
S5	24	Male	AE	+1.00DS/+1.00DC × 90°	0.18
			FE	−0.75DS/−0.50DC × 180°	−0.03
S6	24	Female	AE	+1.50DC × 135°	0.18
			FE	−4.00DS	−0.03

^†^Visual acuity was measured with the Chinese Tumbling E Chart and defined as the log minimum angle of resolution (MAR) associated with 75% correct identification.

**Table 2:** Z-scores of the magnitudes of the measured TAE. SF1 to SF5 represent the five adapting spatial frequency (from low to high) conditions for each observer. All *p* < 0.05

	SF1	SF2	SF3	SF4	SF5
S1	16.79	12.14	4.43	5.41	2.74
S2	9.81	8.84	9.67	1.81	4.00
S3	20.05	10.38	13.06	7.93	7.46
S4	24.80	13.88	2.97	3.67	8.98
S5	26.14	12.95	13.09	3.19	7.27
S6	9.46	4.60	11.39	5.07	4.10
