# Supplementary material for: Tilt after-effect from high spatial-frequency patterns in the amblyopic eye of adults with anisometropic amblyopia
Source: Sci Rep. 2015 Mar 4;5:8728. doi: 10.1038/srep08728 (PMC4348659; doi:10.1038/srep08728)
Supplement: Supplementary Information [file srep08728-s1.pdf]

Tilt after-effect from high spatial-frequency patterns in the amblyopic eye of adults with anisometropic amblyopia

Jiawei Zhou, Lin Li, Pan Zhang, Jie Xi, Yifeng Zhou, Zhong-Lin Lu, Chang-Bing Huang

Inventory

Table S1, related to Figure S1;

Figure S1.

Supplementary Table S1. Characteristics of the observers in the pilot study

Observer ID	Age	Sex	Eye	Correction	Visual acuity† (logMAR)
A1	16	Female	AE	+0.50DS/+0.75DCx75	0.77
			FE	-0.75DS/-0.75DCx10	0.07
A2	16	Female	AE	-2.75DS/-0.50DCx90	0.27
			FE	-2.75DS/-0.37DCx90	0.07
A3	21	Female	LE‡	+5.70DS/+1.50DCx110	0.37
			RE	+5.50DS/+1.50DCx80	0.37
A4	17	Male	AE	+6.00DS/+0.5 DC×90	0.47
			FE	-4.00DS	-0.33
A5	16	Female	AE	- 0.5DS	0.57
			FE	-1.00DS	-0.22

† Visual acuity was measured using the Chinese Tumbling E Chart and defined as the log minimum angle of resolution (MAR) associated with 75% correct identification.

Abbreviations: AE=amblyopic eye; FE=fellow eye; DS=diopters sphere; DC=diopters cylinder; MAR=minimum angle of resolution.

‡ Observer A3 has amblyopia in both eyes, the left eye was used in the test.

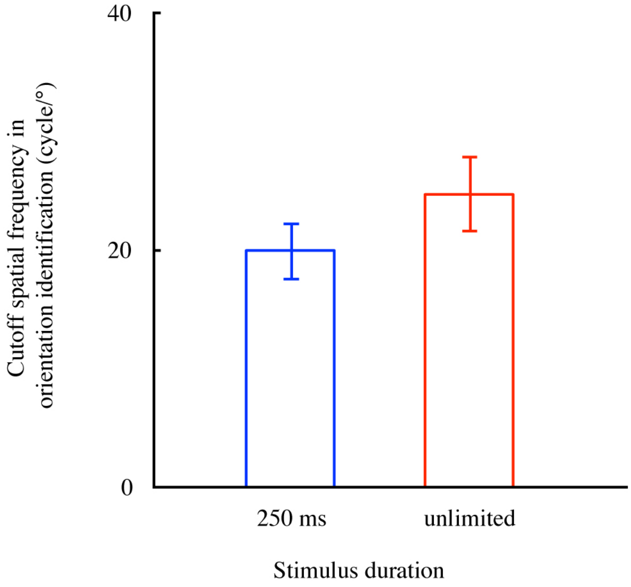

Figure S1. Cutoff spatial frequency in orientation discrimination estimated with 250 ms and unlimited stimulus durations. Results were averaged from five observers, whose clinical details are in Table S1. Note that these five observers only participated in the pilot study but not the main study. Error bars indicate S.E.M. The estimated cutoff spatial frequencies in the two conditions were not significantly different (2-tailed paired sample t-test, $t(4) = -1.25$, $p = 0.28$.)
